# Comparison of local ablative therapies, including radiofrequency ablation, microwave ablation, stereotactic ablative radiotherapy, and particle radiotherapy, for inoperable hepatocellular carcinoma: a systematic review and meta-analysis

**DOI:** 10.1186/s40164-023-00400-7

**Published:** 2023-04-12

**Authors:** Po-Lung Cheng, Ping-Hsiu Wu, We-Yu Kao, Yen-Ting Lai, Jason C. Hsu, Jeng-Fong Chiou, Meng-Huang Wu, Hsin-Lun Lee

**Affiliations:** 1 School of Medicine, College of Medicine, Taipei Medical University, Taipei, Taiwan; 2 Department of Medical Education, Taipei Medical University Hospital, Taipei, Taiwan; 3 Department of Radiation Oncology, Taipei Medical University Hospital, Wuxing street, No. 252, Taipei, 11031 Taiwan; 4 Department of Radiology, School of Medicine, College of Medicine, Taipei Medical University, Taipei, Taiwan; 5 TMU Proton Center, Taipei Medical University, Taipei, Taiwan; 6 Division of Gastroenterology and Hepatology, Department of Internal Medicine, Taipei Medical University Hospital, Taipei, Taiwan; 7 Division of Gastroenterology and Hepatology, Department of Internal Medicine, School of Medicine, College of Medicine, Taipei Medical University, Taipei, Taiwan; 8 TMU Research Center for Digestive Medicine, Taipei Medical University, Taipei, Taiwan; 9 Taipei Cancer Center, Taipei Medical University, Taipei, Taiwan; 10 International PhD Program in Biotech and Healthcare Management, College of Management, Taipei Medical University, Taipei, Taiwan; 11 Clinical Big Data Research Center, Taipei Medical University Hospital, Taipei Medical University, Taipei, Taiwan; 12 Clinical Data Center, Office of Data Science, Taipei Medical University, Taipei, Taiwan; 13 Research Center of Health Care Industry Data Science, College of Management, Taipei Medical University, Taipei, Taiwan; 14 The PhD Program for Translational Medicine, College of Medical Science and Technology, Taipei Medical University and Academia Sinica, Taipei, Taiwan; 15 Spine Division, Department of Orthopedics, Taipei Medical University Hospital, Wuxing street, No. 252, 11031 Taipei, Taiwan; 16 Department of Orthopaedics, School of Medicine, College of Medicine, Taipei Medical University, Taipei, Taiwan; 17 Prospective Innovation Center, Taipei Medical University Hospital, Taipei, Taiwan; 18 TMU Biodesign Center, Taipei Medical University, Taipei, Taiwan

**Keywords:** Inoperable hepatocellular carcinoma, Radiofrequency ablation therapy, Microwave ablation therapy, Stereotactic ablative radiotherapy, Particle radiotherapy, Systematic review, Meta-analysis

## Abstract

Surgical intervention is the first-line treatment in well-selected hepatocellular carcinoma (HCC) patients. However, only a few patients are suitable to receive radical surgery. We conducted a systematic review and meta-analysis to evaluate local control among four local ablative therapies in inoperable HCC patients, including radiofrequency ablation therapy (RFA), microwave ablation therapy (MWA), stereotactic ablative radiotherapy (SABR), and particle radiotherapy. The primary outcome was the local control rate and the secondary were regional and distant progression rates, overall survival rate, and adverse events. We included twenty-six studies from PubMed, EMBASE, and Cochrane Library databases. MWA (p < 0.001) and particle radiotherapy (p < 0.001) showed better performance of local control compared to RFA, while SABR (p = 0.276) showed a non-significant trend. However, SABR (p = 0.002) and particle radiotherapy (p < 0.001) showed better performance than RFA in HCCs of ≥ 30 mm in size. MWA showed a similar result to RFA while SABR and particle radiotherapy showed a lower survival rate in the 2-, 3-, and 4-year overall survival rates. Our results indicate that MWA, SABR and particle radiotherapy were safe and no inferior to RFA in local control rate. Besides, the local control rates of SABR and particle radiotherapy are better than RFA in HCC of ≥ 30 mm in size. As a result, we suggested that MWA, SABR and particle radiotherapy to be effective alternatives to RFA for inoperable HCC. Moreover, the tumor size should be taken into consideration for optimal treatment selection between local ablative therapies.

**Supplementary Information:**

The online version contains supplementary material available at 10.1186/s40164-023-00400-7.

To the Editor

Hepatocellular carcinoma (HCC) is the dominant type of liver cancer, that is the fifth most reported cancers worldwide [1]. Surgery is the primary treatment due to the best outcome in selected patients [2]. However, only 10–37% of patients are suitable for surgery at the time of diagnosis [3]. For inoperable HCC, radiofrequency ablation (RFA) is recommended as the classical local treatment in well-selected patients. Recently, new local ablative modalities, such as microwave ablation therapy (MWA), stereotactic ablative radiotherapy (SABR), and particle radiotherapy show effective and promising results in inoperable HCC; however, their therapeutic efficacy compared to RFA remained unknown because of unavailable comparative prospective trials and inadequate comparison studies. Therefore, we conducted a comprehensive systematic review and meta-analysis to understand the benefit of these local ablative therapies in inoperable HCC.

The definition of inoperable HCC in this study included patients with unresectable HCC, patients who were unable to undergo operation due to physical reasons, and those who were unwilling to receive surgery. The detailed method section was described in Additional file 1. After study selection, 6 randomized controlled trials (RCTs) and 20 non-RCT prospective cohort studies were finally included from PubMed, EMBASE, and Cochrane Library databases (Additional file 2: Fig. S1; Additional file 3: Table S1; Additional file 4). The sensitivity analysis revealed no substantial variations among each publication. The pooled result of local control rate showed that MWA (relative risk (RR): 0.889, 95% confidence interval (CI): 0.852–0.927; p < 0.001) and particle radiotherapy (RR: 0.899, 95% CI: 0.848–0.954; p < 0.001) was statistically higher compared to RFA, while SABR (RR: 0.971, 95% CI: 0.920–1.024; p = 0.276) showed no difference (Table 1; Additional file 5: Fig. S2). The number of single/multiple nodule patients and the different management of the multiple nodule patients are listed, where most of the nodules received fully local treatment (Additional file 3: Table S1). The local control rates of each individual study were listed in the Additional file 6: Table S2. In RFA group, the study by Kan et al. showed a significantly lower local control rate than other RFA studies, which may be because it only included high Child-Pugh class and larger tumor size patients (Additional file 3: Table S1). In SABR group, the study by Bujoid et al. and the study by Scorsetti et al. showed significantly lower local control rates than other SABR studies. It may because that the biologically effective dose of SABR in these two studies was lower than in other SABR studies (Additional file 7: Table S3). Besides, the Child-Pugh class and tumor size were also worse in these two studies than in others (Additional file 3: Table S1). In subgroup analysis, MWA (RR: 0.979, 95% CI: 0.929–1.032; p = 0.427) and SABR (RR: 0.953, 95% CI: 0.884–1.027; p = 0.205) showed similar local control rates in tumor size of < 30 mm in comparison with RFA. However, in tumor size of ≥ 20 mm, MWA (RR: 0.846, 95% CI: 0.799–0.896; p < 0.001), SABR (RR: 0.908, 95% CI: 0.853–0.968; p = 0.003) and particle radiotherapy (RR: 0.860, 95% CI: 0.795–0.930; p < 0.001) had higher local control rate than RFA. In tumor size of ≥ 30 mm, SABR (RR: 0.882, 95% CI: 0.816–0.953; p = 0.002) and particle radiotherapy (RR: 0.805, 95% CI: 0.739–0.877; p < 0.001) had higher local control rate compared with RFA (Table 2).

**Table 1:** Local control rate, regional progression rate and distant progression rate

Groups	Cohorts (n)	Patients (n)	Events (95%)	*I* ^2^	Relative risk (95%)	*p*
Local control rate						
RFA	7	651	0.823 (0.733–0.887)	19.626	1	–
MWA	5	569	0.926 (0.867–0.960)	0.000	0.889 (0.852–0.927)	< 0.001
SABR	7	424	0.848 (0.765–0.906)	38.215	0.971 (0.920–1.024)	0.276
Particle	4	165	0.915 (0.826–0.961)	0.000	0.899 (0.848–0.954)	< 0.001
Regional progression rate						
RFA	3	156	0.298 (0.231–0.375)	0.000	1	–
MWA	2	125	0.136 (0.086–0.208)	0.000	0.456 (0.276–0.755)	0.002
SABR	4	194	0.317 (0.255–0.387)	10.563	1.064 (0.775–1.461)	0.703
Particle	2	71	0.437 (0.327–0.553)	0.000	1.466 (1.026–2.096)	0.036
Distant progression rate						
RFA	4	260	0.064 (0.030–0.132)	9.812	1	–
MWA	2	164	0.024 (0.007–0.083)	0.000	0.375 (0.127–1.105)	0.075
SABR	3	188	0.201 (0.103–0.353)	55.123	3.141 (1.821–5.418)	< 0.001
Particle	2	71	0.187 (0.079–0.379)	0.000	2.922 (1.492–5.720)	0.002

MWA: Microwave ablation; RFA: radiofrequency ablation; SABR: stereotactic ablative radiotherapy

**Table 2:** Subgroup analysis of local control rate

Groups	Cohorts (n)	Patients (n)	Events (95%)	*I* ^2^	Relative risk (95%)	*p*
Local control rate						
≧ 50 mm						
SABR	1	102	0.716 (0.621–0.795)	0.000	1	–
Particle	1	24	0.875 (0.676–0.959)	0.000	0.818 (0.674–0.994)	0.043
< 50 mm						
RFA	7	651	0.822 (0.730–0.888)	16.549	1	–
MWA	5	569	0.926 (0.866–0.960)	0.000	0.888 (0.851–0.926)	< 0.001
SABR	6	322	0.872 (0.787–0.927)	32.839	0.943 (0.892–0.996)	0.035
Particle	2	94	0.909 (0.766–0.968)	0.000	0.904 (0.840–0.973)	0.007
≧ 30 mm						
RFA	3	404	0.724 (0.524–0.862)	7.164	1	–
SABR	5	347	0.821 (0.698–0.901)	25.909	0.882 (0.816–0.953)	0.002
Particle	3	118	0.899 (0.766–0.961)	0.000	0.805 (0.739–0.877)	< 0.001
< 30 mm						
RFA	4	247	0.888 (0.841–0.922)	0.000	1	–
MWA	4	510	0.907 (0.878–0.930)	32.375	0.979 (0.929–1.032)	0.427
SABR	2	77	0.932 (0.846–0.971)	0.000	0.953 (0.884–1.027)	0.205
≧ 20 mm						
RFA	5	468	0.773 (0.636–0.869)	0.000	1	–
MWA	3	412	0.914 (0.819–0.962)	0.000	0.846 (0.799–0.896)	< 0.001
SABR	7	424	0.851 (0.763–0.910)	0.000	0.908 (0.853–0.968)	0.003
Particle	3	118	0.899 (0.775–0.959)	31.931	0.860 (0.795–0.930)	< 0.001
< 20 mm						
RFA	2	183	0.900 (0.847–0.936)	0.000	1	–
MWA	1	98	0.939 (0.870–0.972)	0.000	0.958 (0.894–1.028)	0.234

MWA: Microwave ablation; RFA: radiofrequency ablation; SABR: stereotactic ablative radiotherapy

The pooled result of regional progression rate showed that MWA was significantly lower (RR: 0.456, CI: 0.276–0.755; p = 0.002), particle radiotherapy was higher (RR: 1.466, CI: 1.026–2.096; p = 0.036) and SABR had no difference (RR: 1.064, CI: 0.775–1.461; p = 0.703) compared with RFA (Table 1; Additional file 5: Fig. S3). The pooled result of distant progression rate showed that in comparison with RFA, MWA (RR: 0.375, 95% CI: 0.127–1.105; p = 0.075) was similar while SABR (RR: 3.141, 95% CI: 1.821–5.418; p < 0.001) and particle radiotherapy (RR: 2.922, 95% CI: 1.492–5.720; p = 0.002) had higher rate (Table 1; Additional file 5: Fig. S4). RFA had the highest overall survival rate in all estimated years while MWA did not show a difference, but SABR and particle radiotherapy showed a lower rate (Additional file 5: Fig. S5-7; Additional file 8: Table S4; Additional file 9: Table S5). As for adverse events, we emphasized bleeding, tumor seeding, and abscess in RFA and MWA, as local thermal therapies. Whereas, we emphasized radiation-induced liver disease, dermatitis, and hematologic-related events in SABR and particle radiotherapy, as radiation therapies. None of the events had > 5% of incidence in each arm (Additional file 10: Table S6).

Since the top priority of local ablative therapy was to destroy the small number of abnormal cells and retain the function of the whole organ, the local control rate is the primary outcome to evaluate its efficacy. We included 1,809 participants, which strengthened the evidence of our studies. Our comparison study showed that MWA had an outstanding effect on overall benefit compared with the RFA group. Conversely, two previous meta-analyses before 2016 indicated a similar efficacy between MWA and RFA [4, 5]. Theoretically, MWA heats up more rapidly with a higher temperature than RFA, which may have an advantage of treating more lesions in a shorter time and potentially cause a better therapeutic effect [6]. Our update result corresponded to the design of the technology and reflected the potential benefit of MWA.

The size of HCC might impact the effectiveness of local ablative therapy. Traditionally, RFA, the classic local ablative therapy, is used in HCC of < 30 mm in size while the usage in larger tumors is still controversial [7]. Our study revealed that MWA showed a superior overall local control rate compared to RFA. A previous meta-analysis conducted by Facciorusso et al. revealed that restricted to the patients with high tumor burden (tumor size of > 20 mm), MWA significantly outperformed RFA in local recurrence rate [5]. These results suggested that MWA can be considered as an option to treat larger tumor sizes of HCC.

The evidence for evaluating radiotherapy in HCC treatment remained unclear and is not suggested in the current guidelines because of inadequate prospective studies and especially randomized phase III trials [8, 9]. A previous retrospective study confirmed that both RFA and SABR were able to provide comparable local control rate in inoperable HCC [10]. Nevertheless, our study indicated that particle radiotherapy shows a higher local control rate compared to RFA, and both SABR and particle radiotherapy showed an outstanding effect of local control rate compared with RFA in tumor sizes of ≥ 30 mm. Hence, tumor size is an important factor that related to the local control rate among different local ablative therapies, which should be considered in decision making.

Our study revealed that MWA and particle radiotherapy had a favorable local control rate compared with RFA, and SABR had better local control rate than RFA in tumor size ≥ 30 mm. However, although RFA showed unfavorable local control rate than other therapies, it had a relatively better survival rate when compared to SABR and particle radiotherapy. A previous study revealed that the robust predictors of death in HCC patients included portal vein thrombosis, tumor size, α-fetoprotein level, and C-P classification [11]. In our selected studies, the tumor size in the studies of SABR and particle radiotherapy showed a greater tendency than in the studies of RFA, which is known by analyzing the characterization of each treatment arm in the studies involved in survival rate (Additional file 3: Table S1). Besides, the percentage of patients with C-P classes B and C was also higher in the studies of SABR and particle radiotherapy than in RFA (Additional file 11: Table S7). The difference may be because RFA is the recommended classical local therapy in current guidelines, also the cost of SABR or particle radiotherapy are higher than RFA, which limits the usage as first-line treatment of these therapies in inoperable HCC [12]. The difference in patient selection in these studies is supposed to affect the overall survival rate, which was indicated by the higher regional/distant progression rate and lower survival rate. Further comparative trials should be designed with uniform standards for SABR, particle radiotherapy, and RFA treatments.

Further discussion of the heterogeneity and limitation in this study was described in Additional file 14.

Our results indicate that MWA, SABR, and particle radiotherapy were safe and no inferior to RFA in local control rate. In conclusion, we suggested that MWA, SABR and particle radiotherapy to be effective alternatives to RFA for inoperable HCC. Moreover, the tumor size should be taken into consideration for optimal treatment selection between local ablative therapies.

## Electronic supplementary material

Below is the link to the electronic supplementary material.

Additional file 1: Methods of the study

Additional file 2: Fig. S1. Flowchart of the study selection

Additional file 3: Table S1. Characteristics of selected trials

Additional file 4: Reference of included studies

Additional file 5: Fig. S2. Forest plot for meta-analysis of local control rate

Additional file 6: Table S2. Local control rate of each individual study

Additional file 7: Table S3. Radiotherapy dose regimen of each individual study

Additional file 8: Table S4. 2-, 3-, and 4-year overall survival rate

Additional file 9: Table S5. Subgroup analysis of regional progression rate and 2- and 3-year overall survival rate

Additional file 10: Table S6. Adverse events

Additional file 11: Table S7. Average tumor size and percentage of patient with Child-Pugh class B or above in each arm

Additional file 12: Table S8. Methodological quality assessment of randomized controlled trials

Additional file 13: Table S9. Methodological quality assessment of nonrandomized studies

Additional file 14: Heterogeneity and Limitation

## Data Availability

All data generated or analyzed during this study are included in this published article and its additional files.

## List of abbreviations

CI: Confidence interval
HCC: Hepatocellular carcinoma
MWA: Microwave ablation therapy
RCT: Randomized controlled trials
RFA: Radiofrequency ablation therapy
SABR: Stereotactic ablative radiotherapy
