# Supplementary material for: Comparison of local ablative therapies, including radiofrequency ablation, microwave ablation, stereotactic ablative radiotherapy, and particle radiotherapy, for inoperable hepatocellular carcinoma: a systematic review and meta-analysis
Source: Exp Hematol Oncol. 2023 Apr 12;12:37. doi: 10.1186/s40164-023-00400-7 (PMC10091829; doi:10.1186/s40164-023-00400-7)
Supplement: Supplementary file 1 — Additional file 1: Methods of the study [file 40164_2023_400_MOESM1_ESM.docx]

**Additional file 1.** Methods of the study

The meta-analysis followed the recommendations of the Cochrane Collaboration and the Quality of Reporting of Meta-Analyses PRISMA guidelines (1).

*Selection criteria*

Studies that compared the outcomes of specific intervention to treat inoperable hepatocellular carcinoma (HCC) were included. The inclusion criteria were (1) patients with HCC, with different cancer types in the same study but with distinguishable results from each other; (2) HCC confirmed by pathologic report or radio-imaging study; (3) treatment with radiofrequency ablation therapy, microwave ablation therapy, stereotactic ablative radiotherapy, and particle radiotherapy without the combination of another locoregional treatment, e.g., transplantation and transarterial chemoembolization, and particle radiotherapy included carbon ion and proton therapy; (4) studies with outcomes of local control rate, regional progression rate, or overall survival rate reported at least 2 years; (5) studies including randomized controlled trials (RCT) or prospective cohort studies; (6) inoperable patients, including unresectable HCC, and those unable to underwent operation due to physical reason and are unwilling to receive surgery; (7) studies included patients with extrahepatic metastases did not exclude because the primary outcome was focused on local control rate in this study. All included studies must clearly define treatment procedures. Besides, we only included studies that are published after 2010 (2010 included) because treatment procedures have been advanced in recent years. Studies that aimed to evaluate other interventions are excluded, e.g., single therapy versus combination therapy, and those that evaluate the efficacy of the imaging system, the disease risk factor, and prognostic factors. Additionally, studies with <10 patients or reported recurrent HCC, were published in a non-SCI publication, and were retrospective, phase I trial, letters, case reports, conference abstracts, review, systemic review, and meta-analysis were excluded.

*Search strategy*

Three databases, including PubMed, EMBASE, and Cochrane Library databases were comprehensively searched. Following headings was used in the search: *hepatocellular carcinoma AND stereotactic body radiation therapy OR stereotactic ablative radiotherapy OR proton therapy OR carbon therapy OR particle therapy OR radiofrequency ablation therapy OR microwave ablation therapy*. These termed were searched in full texts. Experts in field identified other articles through manual search of the references. No limitations and other filters were applied. The final search was conducted on March 15, 2022. The study was prospectively registered and accepted by PROSPERO (registration number: CRD42022289787).

*Study selection and data extraction*

The detailed information from each trial was extracted by two reviewers independently, including authors, year of publication, study population characteristics, study design, inclusion and exclusion criteria, intervention techniques, length of follow-up, and outcomes of interest. A third reviewed involved when a disagreement was found between two reviewers.

*Outcome measures*

The primary outcome was the local control rate. The secondary outcomes were the regional progression rate, distant progression rate, survival rate, and adverse event. We assessed the local control rate, regional progression rate, and distant progression rate at the longest duration of complete follow-up. Additionally, we only pooled the results from the reported outcomes for >2 years. Local control rates included those studies that reported local recurrence rates. Regional progression was defined as intra-hepatic progression outside the treated area. Distant progression was defined as extra-hepatic tumor appearance, including solid tumor and lymph node. The adverse events were assessed as the events of grade 3 or higher.

*Methodological quality appraisal*

Two reviewers performed the assessment of each publication independently. A third reviewer involved the quality assessment when a disagreement happened between two reviewers. Risk of bias assessment Version 2 (RoB 2.0) was used to assess the quality of RCT. Five domains of bias was evaluated, including bias arising from the randomization process, deviation from intended intervention, missing outcome data, outcome measurement, and selection reported results. After assessing bias mentioned above, an overall risk of bias was awarded in each trial (2). Meanwhile, Risk of Bias in Nonrandomized Studies of Interventions (ROBIN-I) was used to assess the methodological quality of non-RCT. Three main domains were assessed in ROBIN-I, including bias arising pre-intervention, in intervention, and post-intervention. Different measurements were assessed in each domain, and the overall risk of bias was awarded (3). The assessment of RCT and non-RCT was shown in Additional file 12: Table S8 and Additional file 13: Table S9, respectively.

*Statistical analysis*

Statistical program comprehensive meta-analysis software version 2.0 (Biostat, Englewood, NJ, USA) was applied. Risk ratio was chosen to perform dichotomous data and weight mean difference was reported for continuous outcome. The standard deviation (SD) was calculated using provided CI limits, standard errors, or interquartile ranges (4). The precision of the effect size was reported as 95% confidence interval. Random-effects or fixed models were chosen to be performed according to the heterogeneity, which was reported using the Cochrane Q-test and the *I*^2^ (5,6). Statistically significance was considered if two-sided P-values <0.05. The severity of heterogeneity was considered high, moderate, and low with *I*^2^ of >75%, >50%, and <25%, respectively. *I*^2^ was used across the studies to quantify the proportion of the total outcome variability. A heterogeneity at a high level will undergo sensitivity analysis by omitting one study in turn to estimate the influence of each publication on the outcome. Funnel plot and Egger’s test were used to judge the publication bias (7).

Reference

1. Liberati A, Altman DG, Tetzlaff J, Mulrow C, Gøtzsche PC, Ioannidis JP, et al. The PRISMA statement for reporting systematic reviews and meta-analyses of studies that evaluate health care interventions: explanation and elaboration. Journal of clinical epidemiology. 2009;62(10):e1-34.
2. Higgins JPT TJ, Chandler J, Cumpston M, Li T, Page MJ, Welch VA (editors). Cochrane Handbook for Systematic Reviews of Interventions version 6.3 (updated February 2022). Cochrane, 2022.
3. Sterne JA, Hernán MA, Reeves BC, Savović J, Berkman ND, Viswanathan M, et al. ROBINS-I: a tool for assessing risk of bias in non-randomised studies of interventions. BMJ (Clinical research ed). 2016;355:i4919.
4. Wan X, Wang W, Liu J, Tong T. Estimating the sample mean and standard deviation from the sample size, median, range and/or interquartile range. BMC medical research methodology. 2014; 14: 1-13.
5. Lau J, Ioannidis JP, Schmid CH. Quantitative synthesis in systematic reviews. Ann Intern Med. 1997; 127:820–26.
6. Higgins JP, Thompson SG, Deeks JJ, Altman DG. Measuring inconsistency in meta-analyses. BMJ. 2003; 327:557–60.
