# Supplementary material for: Comparison of local ablative therapies, including radiofrequency ablation, microwave ablation, stereotactic ablative radiotherapy, and particle radiotherapy, for inoperable hepatocellular carcinoma: a systematic review and meta-analysis
Source: Exp Hematol Oncol. 2023 Apr 12;12:37. doi: 10.1186/s40164-023-00400-7 (PMC10091829; doi:10.1186/s40164-023-00400-7)
Supplement: Supplementary file 2 — Additional file 2: Fig. S1. Flowchart of the study selection [file 40164_2023_400_MOESM2_ESM.docx]

**Additional file 2: Fig. S1** Flowchart of the study selection

Studies included in qualitative synthesis

(meta-analysis)

(n = 26)

Full-text articles assessed for eligibility

(n = 785)

Records screened

(n = 18,249)

Full-text articles excluded, with reasons (total n = 759):

- Duplicate series (n = 8)

- Improper patients (n = 92)

- Different interventions (n = 168)

- Inappropriate study type (n = 391)

- Non-relevant data (n = 100)

Records excluded

(n = 17464)

Records after duplicate removed

(n = 18,249)

Records identified through database searching (n = 24,635)

- Pubmed (n = 7,730)

- Embase (n = 16,356)

- Cochrane library (n = 549)
