# Supplementary material for: Comparison of local ablative therapies, including radiofrequency ablation, microwave ablation, stereotactic ablative radiotherapy, and particle radiotherapy, for inoperable hepatocellular carcinoma: a systematic review and meta-analysis
Source: Exp Hematol Oncol. 2023 Apr 12;12:37. doi: 10.1186/s40164-023-00400-7 (PMC10091829; doi:10.1186/s40164-023-00400-7)
Supplement: Supplementary file 3 — Additional file 3: Table S1. Characteristics of selected trials [file 40164_2023_400_MOESM3_ESM.doc]

| **Additional file 3: Table S1** Characteristics of selected trials | | | | | | | | | | |
| --- | --- | --- | --- | --- | --- | --- | --- | --- | --- | --- |
| Study | Study design | Intervention | No. of HCC patients  (male %) | Age (years),  median (range) | Staging (BCLC) | Tumor size:  Median ± range (mm) | Child-Pugh class  (A/B/C) | Patient with single or multiple nodules | Different management of multiple nodules | Median of follow-up |
| Abdelaziz et al. [2014] | RCT | MWA | 66 (73) | 53.6 ± 5^§^ | A: 66 | 29 ± 9.7^§^ | 25/41/0 | Single: 57  Multiple: 9 | No mention | Unknown |
|  |  | RFA | 45 (69) | 56.8 ± 7.3^§^ | A: 45 | 29.5 ± 10.3^§^ | 24/21/0 | Single: 37  Multiple: 8 | No mention |  |
| Bujold et al. [2013] | Prospective | SABR | 102 (78) | 69.4 (40.4-90.3) | A/B: 35  C: 67 | 72 (14-231) | 102/0/0 | Single: 40  Multiple: 62 | Some multiple nodules cases received a lower dose to meet planning objectives | 31.4 m |
| Choi et al. [2016] | Prospective | RFA | 79 (67) | 61.7 ± 9.1^§^ | Unknown | 19 ± 7^§^ | 73/6/0 | Single: 63  Multiple: 16 | All nodules are treated as the single nodule protocol | Range: 22.4-42.7 m |
| Chong et al. [2020] | RCT | MWA | 47 (64) | 63 (50-80) | Unknown | 31 (20-45) | 39/7/1 | Unknown | No mention | 38.3 m (2.3-78)^＃^ |
|  |  | RFA | 46 (83) | 64.5 (42-85) | Unknown | 28 (20-55) | 40/6/0 | Unknown |  | 33.9 m (4.9-72.7)^＃^ |
| Cillo et al. [2014] | Prospective | MWA | 42 (83) | 64 (47–81) | 0: 10  A: 23  B: 9 | 25 (15–53) | 28/14/0 | Single: 22  Multiple: 20 | No mention | Minimal follow-up 24 m |
| Darweesh et al. [2019] | Prospective | MWA | 59 (75) | 57.2 ± 6^§^ | Unknown | < 30: 44  30-50: 15 | 22/37/0 | Single: 53  Multiple: 6 | All nodules are treated as the single nodule protocol | 34 m^§^ |
| Durand-Labrunie et al. [2020] | Prospective | SABR | 43 (81) | 72 (43-91) | Unknown | 28 (10-60) | 37/5/0  1 unknown | Single: 43  Multiple: 0 | No mention | 4 yr (1.2-4.6) |
| Feng et al. [2018] | Prospective | SABR | 69 | 62 (34-85)^◇^ | Unknown | 30 (0-130)^◇^ | Median score: 6  Range: 5-9 | Single: 51^◇^  Multiple: 18^◇^ | All nodules are treated as the single nodule protocol | 37 m |
| Francica et al. [2019] | Prospective | RFA | 25 (36) | 76 (61-84) | Unknown | 40 (32-55) | 22/3/0 | Single: 24  Multiple: 1 | No mention | 30 m (6-57)^＃^ |
| Imada et al. [2010] | Prospective | Carbon | 64 (75) | 69 (37-84) | Unknown | 40 (12-120) | 49/15/0 | Single: 56  Multiple: 8 | All nodules are treated as the single nodule protocol | Uncertain |
| Kan et al. [2015] | RCT | RFA | 32 (78) | 52.4 ± 8.9^§^ | A: 0  B: 14  C: 18 | Range: 31-50 | 14/18/0 | Single: 32  Multiple: 0 | N/A | Unknown |
| Kimura et al.[2017] | Prospective | Proton | 24 (88) | 73 (49-89) | Unknown | 50-100: 13  ≧ 100: 11 | 24/0/0 | Single: 24  Multiple: 0 | N/A | 17.5 m (3-64)^＃^ |
| Kimura et al. [2021] | Prospective | SABR | 36 (64) | 73.5 (57-85) | 0: 12  A: 16  C: 8 | 23 (10-50) | 33/3/0 | Unknown | No mention | 21 m (4-57)^＃^ |
| Lasley et al. [2015] | Prospective | SABR | 59 (80) | 61 (24-86) | A: 38  B: 21 | 33.6 cc (2-107.3) | 38/21/0 | Single: 48  Multiple: 11 | All nodules are treated as the single nodule protocol | 33.3 m (2.8-61.1)^＃^ |
| Liu et al. [2017] | Prospective | SABR | 74 (76) | > 60 y: 34  ≦ 60 y: 40 | 0: 15  A: 59 | 31.08 ± 27.69^§^ cc | 74/0/0 | Unknown | Multifocal lesions were restricted to a maximum of three lesions with a maximum lesion size of 3 cm for each lesion. | 2 yr |
| Nakayama et al. [2011] | Prospective | Proton | 47 (66) | 69 (43-82) | Unknown | Unknown | 35/9/3 | Unknown | No mention | 23 m (2.8-52.4)^＃^ |
| Nojiri et al. [2017] | RCT | RFA | 26 (58) | 69.1 ± 11^§^ | Unknown | 17.8 (6-30) | 23/3/0 | 1.3 (1-3)^＃^ | No mention | 3.9 yr (736-1818 d) |
| Parzen et al. [2020] | Prospective | Proton | 30 (73) | 70.5 (34–89) | Unknown | 43 (12–94) | Unknown | Unknown | No mention | 5.1 m (0.1-40.8)^＃^ |
| Scorsetti et al. [2015] | Prospective | SABR | 43 (72) | 72 (46-87) | A: 10  B: 15  C: 9 | 48 (10-125) | 23/20/0 | Single: 24  Multiple: 19 | All nodules are treated as single nodule protocol | 8 m (3-43)^＃^ |
| Tak et al. [2018] | RCT | RFA | 347 (76) | 18-64: 207  > 65: 138  Missing: 2 | A: 219  B: 116  missing: 12 | 30-50: 286  > 50-70: 61 | 329/18/0 | Single: 219  Multiple: 128 | No mention | Unknown |
| Violi et al. [2018] | RCT | MWA | 71 (83) | 68 (60-72) | 0: 8  A: 63 | 18 ± 6.5^§^ | 57/14/0 | Single: 44  Multiple: 24 | All nodules are treated as single nodule protocol | 26 m (18-29)* |
|  |  | RFA | 73 (85) | 65 (59-73) | 0: 11  A: 62 | 18 ± 7.1^§^ | 53/20/0 | Single: 46  Multiple: 26 |  | 25 m (18-34)* |
| Wang et al. [2010] | Prospective | RFA | 12 (83) | 58 ± 8.8^§^ | Unknown | 24 ± 5^§^ | 12/0/0 | Single: 11  Multiple: 1 | All nodules are treated as single nodule protocol | 28 m ± 13.2^§^ |
| Weiner et al. [2016] | Prospective | SABR | 12 | 72 (51-95)^◇^ | A: 5  B: 4  C: 3 | 50 (16-123)^◇^ | 23/3/0^◇^ | Single: 22^◇^  Multiple: 4^◇^ | All nodules are treated as single nodule protocol | 8.8 m (0.3-33)^＃^ |
| Yao et al. [2021] | Prospective | RFA | 48 (92) | 58 (37-89) | A: 18  B: 40 | 32 (12-66) | 48/0/0 | Unknown | No mention | 25.6 m (13.5-35.2)^＃^ |
| Yu et al. [2018] | Prospective | Proton | 101 (86) | 63 (35-91) | Unknown | 25 (10-160) | 90/10/1 | Single: 74  Multiple: 27 | At least one patient was only treated in dominant lesion due to large multiple nodules (>14 cm) | 4.9 m (1.3-14.6)^＃^ |
| Zhou et al. [2011] | Prospective | MWA | 215 (87) | 57.3 ± 11^§^ | Unknown | 29 (11-60) | 97/109/9 | Single: 151  Multiple: 64 | No mention | Unknown |
|  | BCLC: Barcelona Clinic Liver Cancer classification; d: days; m: months; MWA: microwave ablation; RCT: randomized controlled trial; RFA: radiofrequency ablation; SABR: stereotactic ablative radiotherapy; SD: standard deviation; yr: years  ^§^: mean ± SD  ^＃^: median (range)  *: median (IQR)  ^◇^: unable to separate from other tumor type or wrong intervention | | | | | | | | | |
