# Supplementary material for: Comparison of local ablative therapies, including radiofrequency ablation, microwave ablation, stereotactic ablative radiotherapy, and particle radiotherapy, for inoperable hepatocellular carcinoma: a systematic review and meta-analysis
Source: Exp Hematol Oncol. 2023 Apr 12;12:37. doi: 10.1186/s40164-023-00400-7 (PMC10091829; doi:10.1186/s40164-023-00400-7)
Supplement: Supplementary file 4 — Additional file 4: Reference of included studies [file 40164_2023_400_MOESM4_ESM.docx]

**Additional file 4:** Reference of included studies

1. Abdelaziz A, Elbaz T, Shousha HI, Mahmoud S, Ibrahim M, Abdelmaksoud A, et al. Efficacy and survival analysis of percutaneous radiofrequency versus microwave ablation for hepatocellular carcinoma: an Egyptian multidisciplinary clinic experience. Surg Endosc. 2014;28(12):3429-34.
2. Bujold A, Massey CA, Kim JJ, Brierley J, Cho C, Wong RK, et al. Sequential phase I and II trials of stereotactic body radiotherapy for locally advanced hepatocellular carcinoma. J Clin Oncol. 2013;31(13):1631-9.
3. Choi JW, Lee JM, Lee DH, Yoon JH, Suh KS, Yoon JH, et al. Switching Monopolar Radiofrequency Ablation Using a Separable Cluster Electrode in Patients with Hepatocellular Carcinoma: A Prospective Study. PLoS One. 2016;11(8):e0161980.
4. Chong CCN, Lee KF, Cheung SYS, Chu CCM, Fong AKW, Wong J, et al. Prospective double-blinded randomized controlled trial of Microwave versus RadioFrequency Ablation for hepatocellular carcinoma (McRFA trial). HPB (Oxford). 2020;22(8):1121-7.
5. Cillo U, Noaro G, Vitale A, Neri D, D'Amico F, Gringeri E, et al. Laparoscopic microwave ablation in patients with hepatocellular carcinoma: a prospective cohort study. HPB (Oxford). 2014;16(11):979-86.
6. Darweesh SK, Gad AA. Percutaneous microwave ablation for HCV-related hepatocellular carcinoma: Efficacy, safety, and survival. Turk J Gastroenterol. 2019;30(5):445-53.
7. Durand-Labrunie J, Baumann AS, Ayav A, Laurent V, Boleslawski E, Cattan S, et al. Curative Irradiation Treatment of Hepatocellular Carcinoma: A Multicenter Phase 2 Trial. Int J Radiat Oncol Biol Phys. 2020;107(1):116-25.
8. Feng M, Suresh K, Schipper MJ, Bazzi L, Ben-Josef E, Matuszak MM, et al. Individualized Adaptive Stereotactic Body Radiotherapy for Liver Tumors in Patients at High Risk for Liver Damage: A Phase 2 Clinical Trial. JAMA Oncol. 2018;4(1):40-7.
9. Francica G, Altiero M, Laccetti E, Pezzullo F, Tanga M, Avitabile G, et al. Long-term follow-up of unresectable medium-large hepatocellular carcinoma nodules treated with radiofrequency ablation using a multiple-electrode switching system. The British journal of radiology. 2019;92(1093):20180625.
10. Imada H, Kato H, Yasuda S, Yamada S, Yanagi T, Kishimoto R, et al. Comparison of efficacy and toxicity of short-course carbon ion radiotherapy for hepatocellular carcinoma depending on their proximity to the porta hepatis. Radiother Oncol. 2010;96(2):231-5.
11. Kan X, Jing Y, Wan QY, Pan JC, Han M, Yang Y, et al. Sorafenib combined with percutaneous radiofrequency ablation for the treatment of medium-sized hepatocellular carcinoma. European review for medical and pharmacological sciences. 2015;19(2):247-55.
12. Kimura K, Nakamura T, Ono T, Azami Y, Suzuki M, Wada H, et al. Clinical results of proton beam therapy for hepatocellular carcinoma over 5 cm. Hepatol Res. 2017;47(13):1368-74.
13. Kimura T, Takeda A, Sanuki N, Ariyoshi K, Yamaguchi T, Imagumbai T, et al. Multicenter prospective study of stereotactic body radiotherapy for previously untreated solitary primary hepatocellular carcinoma: The STRSPH study. Hepatol Res. 2021;51(4):461-71.
14. Lasley FD, Mannina EM, Johnson CS, Perkins SM, Althouse S, Maluccio M, et al. Treatment variables related to liver toxicity in patients with hepatocellular carcinoma, Child-Pugh class A and B enrolled in a phase 1-2 trial of stereotactic body radiation therapy. Pract Radiat Oncol. 2015;5(5):e443-e9.
15. Liu X, Song Y, Liang P, Su T, Zhang H, Zhao X, et al. Analysis of the factors affecting the safety of robotic stereotactic body radiation therapy for hepatocellular carcinoma patients. Onco Targets Ther. 2017;10:5289-95.
16. Nakayama H, Sugahara S, Fukuda K, Abei M, Shoda J, Sakurai H, et al. Proton beam therapy for hepatocellular carcinoma located adjacent to the alimentary tract. Int J Radiat Oncol Biol Phys. 2011;80(4):992-5.
17. Nojiri S, Fujiwara K, Shinkai N, Iio E, Joh T. Effects of branched-chain amino acid supplementation after radiofrequency ablation for hepatocellular carcinoma: A randomized trial. Nutrition. 2017;33:20-7.
18. Parzen JS, Hartsell W, Chang J, Apisarnthanarax S, Molitoris J, Durci M, et al. Hypofractionated proton beam radiotherapy in patients with unresectable liver tumors: multi-institutional prospective results from the Proton Collaborative Group. Radiat Oncol. 2020;15(1):255.
19. Scorsetti M, Comito T, Cozzi L, Clerici E, Tozzi A, Franzese C, et al. The challenge of inoperable hepatocellular carcinoma (HCC): results of a single-institutional experience on stereotactic body radiation therapy (SBRT). J Cancer Res Clin Oncol. 2015;141(7):1301-9.
20. Tak WY, Lin SM, Wang Y, Zheng J, Vecchione A, Park SY, et al. Phase III HEAT Study Adding Lyso-Thermosensitive Liposomal Doxorubicin to Radiofrequency Ablation in Patients with Unresectable Hepatocellular Carcinoma Lesions. Clin Cancer Res. 2018;24(1):73-83.
21. Vietti Violi N, Duran R, Guiu B, Cercueil J-P, Aubé C, Digklia A, et al. Efficacy of microwave ablation versus radiofrequency ablation for the treatment of hepatocellular carcinoma in patients with chronic liver disease: a randomised controlled phase 2 trial. The Lancet Gastroenterology & Hepatology. 2018;3(5):317-25.
22. Wang JH, Tung HD, Chen TY, Hung CH, Chen CH, Changchien CS, et al. Radiofrequency ablation of small hepatocellular carcinoma with intravenous pegylated liposomal doxorubicin. Hepatol Int. 2010;5(1):567-74.
23. Weiner AA, Olsen J, Ma D, Dyk P, DeWees T, Myerson RJ, et al. Stereotactic body radiotherapy for primary hepatic malignancies - Report of a phase I/II institutional study. Radiother Oncol. 2016;121(1):79-85.
24. Yao XS, Yan D, Jiang XX, Li X, Zeng HY, Li H. Short-term outcomes of radiofrequency ablation for hepatocellular carcinoma using cone-beam computed tomography for planning and image guidance. World J Clin Cases. 2021;9(7):1580-91.
25. Yu JI, Yoo GS, Cho S, Jung SH, Han Y, Park S, et al. Initial clinical outcomes of proton beam radiotherapy for hepatocellular carcinoma. Radiat Oncol J. 2018;36(1):25-34.
26. Zhou P, Liang P, Dong B, Yu X, Han X, Wang Y, et al. Long-term results of a phase II clinical trial of superantigen therapy with staphylococcal enterotoxin C after microwave ablation in hepatocellular carcinoma. Int J Hyperthermia. 2011;27(2):132-9.
