# Supplementary material for: Comparison of local ablative therapies, including radiofrequency ablation, microwave ablation, stereotactic ablative radiotherapy, and particle radiotherapy, for inoperable hepatocellular carcinoma: a systematic review and meta-analysis
Source: Exp Hematol Oncol. 2023 Apr 12;12:37. doi: 10.1186/s40164-023-00400-7 (PMC10091829; doi:10.1186/s40164-023-00400-7)
Supplement: Supplementary file 5 — Additional file 5: Fig. S2. Forest plot for meta-analysis of local control rate [file 40164_2023_400_MOESM5_ESM.docx]

**Additional file 5:**

**Fig. S2** Forest plot for meta-analysis of local control rate

**Fig. S3** Forest plot for meta-analysis of regional progression rate

**Fig. S4** Forest plot for meta-analysis of distant progression rate

**Fig. S5** Forest plot for meta-analysis of 2-year overall survival rate

**Fig. S6** Forest plot for meta-analysis of 3-year overall survival rate

**Fig. S7** Forest plot for meta-analysis of 4-year overall survival rate
