# Supplementary material for: Comparison of local ablative therapies, including radiofrequency ablation, microwave ablation, stereotactic ablative radiotherapy, and particle radiotherapy, for inoperable hepatocellular carcinoma: a systematic review and meta-analysis
Source: Exp Hematol Oncol. 2023 Apr 12;12:37. doi: 10.1186/s40164-023-00400-7 (PMC10091829; doi:10.1186/s40164-023-00400-7)
Supplement: Supplementary file 6 — Additional file 6: Table S2. Local control rate of each individual study [file 40164_2023_400_MOESM6_ESM.docx]

**Additional file 6: Table S2** Local control rate of each individual study

| Study | Intervention | Local control rate |
| --- | --- | --- |
| Abdelaziz et al. [2014] | RFA | 2-year: 0.865 |
| Violi et al. [2018] | RFA | 2-year: 0.885 |
| Choi et al. [2016] | RFA | 2-year: 0.924 |
| Francia et al. [2019] | RFA | **3-year: 0.72*** |
| Kan et al. [2015] | RFA | **3-year: 0.531*** |
| Tak et al. [2018] | RFA | 3-year: 0.841 |
| Wang et al. [2011] | RFA | 3-year: 0.833 |
| Abdelaziz et al. [2014] | MWA | **2-year: 0.961*** |
| Cillo et al. [2014] | MWA | 2-year: 0.881 |
| Darweesh et al. [2019] | MWA | 2-year: 0.949 |
| Violi et al. [2018] | MWA | 2-year: 0.939 |
| Zhou et al. [2011] | MWA | 5-year: 0.895 |
| Bujoid et al. [2013] | SABR | **3-year: 0.716*** |
| Durand-Labrunie et al. [2020] | SABR | **2-year: 0.953*** |
| Feng et al. [2017] | SABR | **2-year: 0.957*** |
| Kimura et al. [2020] | SABR | **3-year: 0.912*** |
| Lasley et al. [2015] | SABR | 3-year: 0.881 |
| Liu et al. [2017] | SABR | 2-year: 0.824 |
| Scorsetti et al. [2015] | SABR | **2-year: 0.651*** |
| Imada et al. [2010] | Particle | 5-year: 0.937 |
| Kimura et al. [2017] | Particle | 2-year: 0.875 |
| Nakayama et al. [2011] | Particle | 4-year: 0.957 |
| Parzen et al. [2020] | Particle | 2-year: 0.867 |

***: value outside of 95% confidence interval**
