# Supplementary material for: Comparison of local ablative therapies, including radiofrequency ablation, microwave ablation, stereotactic ablative radiotherapy, and particle radiotherapy, for inoperable hepatocellular carcinoma: a systematic review and meta-analysis
Source: Exp Hematol Oncol. 2023 Apr 12;12:37. doi: 10.1186/s40164-023-00400-7 (PMC10091829; doi:10.1186/s40164-023-00400-7)
Supplement: Supplementary file 7 — Additional file 7: Table S3. Radiotherapy dose regimen of each individual study [file 40164_2023_400_MOESM7_ESM.docx]

| **Additional file 7: Table S3** Radiotherapy dose regimen of each individual study | | | |
| --- | --- | --- | --- |
| Study | Intervention | Radiotherapy dose regimen | Biologically effective dose (BED10) |
| Bujoid et al. [2013] | SABR | 24-54 Gy/6 FXs | 33.6-102.6 |
| Durand-Labrunie et al. [2020] | SABR | 45 Gy/3 FXs | 112.5 |
| Feng et al. [2017] | SABR | 23-60 Gy/3-5 FXs | 33.58-18 |
| Kimura et al. [2020] | SABR | 40 Gy/5 FXs | 72 |
| Lasley et al. [2015] | SABR | Child A: 48 Gy/3 FXs | 124.8 |
|  |  | Child B: 40 Gy/5 FXs | 72 |
| Liu et al. [2017] | SABR | 45 Gy/3 FXs | 112.5 |
| Scorsetti et al. [2015] | SABR | Lesions <3 cm: 48-75 Gy/3 FXs | 124.8-262.5 |
|  |  | Lesions >3 cm & < 6 cm: 36-60 Gy/6 FXs | 57.6-120 |
| Weiner et al.[2016] | SABR | 40-55 Gy/5 FXs | 72-115.5 |
| Imada et al. [2010] | Particle (Carbon) | 52.8 GyE/4 FXs | 122.496 |
| Kimura et al. [2017] | Particle (Proton) | 60.8-85.8 GyE /22 FXs | 77.60-119.26 |
| Nakayama et al. [2011] | Particle (Proton) | Early 16 patients: 72.6 GyE/22 FX | 96.56 |
|  |  | Late 31 patients: 77 GyE/35 FXs | 93.94 |
| Parzen et al. [2020] | Particle (Proton) | 32.5-50 GyE/5 FXs or  45-67.5 GyE/15 FXs | 53.63-100 (5FXs) or  58.5-97.875 (15FXs) |
| Yu et al.[2018] | Particle (Proton) | 62-92 GyE/10 FXs | 100.44-176.64 |

Gy: Gray; FXs: fractions; GyE: Gray equivalent; Formula of BED10 = total dose x (1 + dose per fraction/α/β), α/β=10.
