# Supplementary material for: Comparison of local ablative therapies, including radiofrequency ablation, microwave ablation, stereotactic ablative radiotherapy, and particle radiotherapy, for inoperable hepatocellular carcinoma: a systematic review and meta-analysis
Source: Exp Hematol Oncol. 2023 Apr 12;12:37. doi: 10.1186/s40164-023-00400-7 (PMC10091829; doi:10.1186/s40164-023-00400-7)
Supplement: Supplementary file 8 — Additional file 8: Table S4. 2-, 3-, and 4-year overall survival rate [file 40164_2023_400_MOESM8_ESM.docx]

| **Additional file 8: Table S4** 2-, 3-, and 4-year overall survival rate | | | | | | |
| --- | --- | --- | --- | --- | --- | --- |
| Groups | Cohorts (n) | Patients (n) | Events (95%) | *I*^2^ | Relative risk (95%) | *p* |
| 2-year OS |  |  |  |  |  |  |
| RFA | 8 | 602 | 0.785 (0.664–0.871) | 0.000 | 1 | – |
| MWA | 6 | 462 | 0.779 (0.646–0.872) | 0.000 | 1.008 (0.945–1.074) | 0.814 |
| SABR | 7 | 424 | 0.660 (0.512–0.782) | 48.578 | 1.189 (1.098–1.289) | <0.001 |
| Particle | 4 | 165 | 0.560 (0.357–0.744) | 53.296 | 1.402 (1.217–1.615) | <0.001 |
| 3-year OS |  |  |  |  |  |  |
| RFA | 6 | 529 | 0.738 (0.627–0.825) | 0.000 | 1 | – |
| MWA | 3 | 333 | 0.726 (0.582–0.835) | 0.000 | 1.017 (0.935–1.105) | 0.700 |
| SABR | 5 | 248 | 0.483 (0.356–0.612) | 55.869 | 1.528 (1.330–1.755) | <0.001 |
| Particle | 3 | 135 | 0.539 (0.373–0.696) | 0.000 | 1.369 (1.162–1.613) | <0.001 |
| 4-year OS |  |  |  |  |  |  |
| RFA | 4 | 444 | 0.575 (0.445–0.696) | 0.000 | 1 | – |
| MWA | 2 | 262 | 0.573 (0.405–0.726) | 0.000 | 1.003 (0.880–1.145) | 0.959 |
| SABR | 3 | 146 | 0.329 (0.207–0.480) | 75.245 | 1.748 (1.368–2.233) | <0.001 |
| Particle | 3 | 135 | 0.330 (0.208–0.479) | 0.000 | 1.742 (1.353–2.245) | <0.001 |
| MWA: Microwave ablation; OS: overall survival; RFA: radiofrequency ablation; SABR: stereotactic ablative radiotherapy | | | | | | |
