# Supplementary material for: Comparison of local ablative therapies, including radiofrequency ablation, microwave ablation, stereotactic ablative radiotherapy, and particle radiotherapy, for inoperable hepatocellular carcinoma: a systematic review and meta-analysis
Source: Exp Hematol Oncol. 2023 Apr 12;12:37. doi: 10.1186/s40164-023-00400-7 (PMC10091829; doi:10.1186/s40164-023-00400-7)
Supplement: Supplementary file 9 — Additional file 9: Table S5. Subgroup analysis of regional progression rate and 2- and 3-year overall survival rate [file 40164_2023_400_MOESM9_ESM.docx]

| **Additional file 9: Table S5** Subgroup analysis of regional progression rate and 2- and 3-year overall survival rate | | | | | | |
| --- | --- | --- | --- | --- | --- | --- |
| Groups | Cohorts (n) | Patients (n) | Events (95%) | *I*^2^ | Relative risk (95%) | *p* |
| Regional progression rate |  |  |  |  |  |  |
| ≧50 mm |  |  |  |  |  |  |
| Particle | 1 | 24 | 0.417 (0.241–0.617) | 0.000 | – | – |
| < 50 mm |  |  |  |  |  |  |
| RFA | 3 | 156 | 0.298 (0.231–0.375) | 0.000 | 1 | – |
| MWA | 2 | 125 | 0.136 (0.086–0.208) | 0.000 | 0.456 (0.276–0.755) | 0.002 |
| SABR | 4 | 194 | 0.317 (0.255–0.387) | 10.563 | 1.064 (0.775–1.461) | 0.703 |
| ≧30 mm |  |  |  |  |  |  |
| RFA | 1 | 32 | 0.281 (0.132–0.502) | 0.000 | 1 | – |
| SABR | 2 | 117 | 0.324 (0.216–0.455) | 0.000 | 1.153 (0.625–2.128) | 0.649 |
| Particle | 1 | 24 | 0.417 (0.211–0.656) | 0.000 | 1.484 (0.716–3.075) | 0.288 |
| <30 mm |  |  |  |  |  |  |
| RFA | 3 | 156 | 0.295 (0.223–0.379) | 0.000 | 1 | – |
| MWA | 1 | 66 | 0.136 (0.070–0.249) | 0.000 | 0.461 (0.240–0.887) | 0.020 |
| SABR | 2 | 77 | 0.315 (0.216–0.435) | 20.470 | 1.068 (0.709–1.607) | 0.753 |
| ≧20 mm |  |  |  |  |  |  |
| RFA | 2 | 77 | 0.248 (0.164–0.356) | 0.000 | 1 | – |
| MWA | 1 | 66 | 0.136 (0.073–0.242) | 0.000 | 0.548 (0.266–1.129) | 0.103 |
| SABR | 4 | 194 | 0.317 (0.255–0.387) | 10.563 | 1.278 (0.823–1.985) | 0.275 |
| Particle | 1 | 24 | 0.417 (0.211–0.656) | 0.000 | 1.681 (0.911–3.102) | 0.096 |
| <20 mm |  |  |  |  |  |  |
| RFA | 1 | 79 | 0.342 (0.246–0.453) | 0.006 | – | – |
| 2-year OS |  |  |  |  |  |  |
| ≧50 mm |  |  |  |  |  |  |
| SABR | 1 | 102 | 0.343 (0.023–0.921) | 0.000 | 1 | – |
| Particle | 1 | 24 | 0.542 (0.047–0.966) | 0.000 | 0.633 (0.401–0.998) | 0.049 |
| <50 mm |  |  |  |  |  |  |
| RFA | 8 | 602 | 0.785 (0.664–0.870) | 0.000 | 1 | – |
| MWA | 6 | 462 | 0.779 (0.647–0.872) | 0.000 | 1.008 (0.945–1.074) | 0.814 |
| SABR | 6 | 322 | 0.713 (0.561–0.828) | 40.996 | 1.101 (1.015–1.194) | 0.020 |
| Particle | 2 | 94 | 0.561 (0.282–0.806) | 84.618 | 1.399 (1.165–1.681) | < 0.001 |
| ≧30 mm |  |  |  |  |  |  |
| RFA | 3 | 420 | 0.793 (0.536–0.927) | 0.000 | 1 | – |
| MWA | 1 | 47 | 0.809 (0.342–0.972) | 0.000 | 0.980 (0.846–1.136) | 0.790 |
| SABR | 5 | 347 | 0.618 (0.392–0.802) | 33.884 | 1.283 (1.166–1.413) | <0.001 |
| Particle | 3 | 118 | 0.549 (0.264–0.805) | 50.584 | 1.444 (1.218–1.713) | <0.001 |
| <30 mm |  |  |  |  |  |  |
| RFA | 5 | 182 | 0.773 (0.633–0.870) | 40.729 | 1 | – |
| MWA | 4 | 356 | 0.793 (0.665–0.880) | 0.000 | 0.975 (0.886–1.072) | 0.598 |
| SABR | 2 | 77 | 0.776 (0.564–0.903) | 0.000 | 0.996 (0.863–1.150) | 0.958 |
| ≧20 mm |  |  |  |  |  |  |
| RFA | 6 | 503 | 0.747 (0.582–0.863) | 0.000 | 1 | – |
| MWA | 4 | 332 | 0.775 (0.585–0.894) | 0.000 | 0.964 (0.892–1.041) | 0.350 |
| SABR | 7 | 424 | 0.663 (0.501–0.794) | 37.958 | 1.127 (1.035–1.226) | 0.006 |
| Particle | 3 | 118 | 0.552 (0.299–0.781) | 62.776 | 1.353 (1.141–1.605) | < 0.001 |
| <20 mm |  |  |  |  |  |  |
| RFA | 2 | 99 | 0.894 (0.663–0.973) | 0.000 | 1 | – |
| MWA | 1 | 71 | 0.859 (0.510–0.973) | 0.000 | 1.041 (0.927–1.169) | 0.500 |
| 3-year OS |  |  |  |  |  |  |
| ≧50 mm |  |  |  |  |  |  |
| Particle | 1 | 24 | 0.542 (0.346–0.725) | 0.000 | – | – |
| <50 mm |  |  |  |  |  |  |
| RFA | 6 | 529 | 0.741 (0.621–0.833) | 0.000 | 1 | – |
| MWA | 3 | 333 | 0.727 (0.570–0.843) | 0.000 | 1.019 (0.938–1.107) | 0.652 |
| SABR | 5 | 248 | 0.485 (0.349–0.623) | 49.323 | 1.528 (1.331–1.754) | < 0.001 |
| Particle | 1 | 64 | 0.563 (0.279–0.811) | 0.000 | 1.316 (1.055–1.643) | 0.015 |
| ≧30 mm |  |  |  |  |  |  |
| RFA | 2 | 372 | 0.640 (0.480–0.774) | 0.000 | 1 | – |
| MWA | 1 | 47 | 0.681 (0.442–0.852) | 0.000 | 0.940 (0.762–1.159) | 0.562 |
| SABR | 3 | 171 | 0.377 (0.258–0.513) | 46.504 | 1.698 (1.380–2.088) | < 0.001 |
| Particle | 2 | 88 | 0.554 (0.379–0.717) | 0.000 | 1.155 (0.944–1.414) | 0.162 |
| <30 mm |  |  |  |  |  |  |
| RFA | 4 | 157 | 0.798 (0.661–0.889) | 0.000 | 1 | – |
| MWA | 2 | 286 | 0.746 (0.564–0.870) | 17.532 | 1.070 (0.964–1.187) | 0.203 |
| SABR | 2 | 77 | 0.665 (0.445–0.830) | 41.180 | 1.200 (1.005–1.432) | 0.043 |
| ≧20 mm |  |  |  |  |  |  |
| RFA | 4 | 430 | 0.677 (0.540–0.789) | 0.000 | 1 | – |
| MWA | 2 | 262 | 0.669 (0.496–0.806) | 0.000 | 1.012 (0.909–1.127) | 0.828 |
| SABR | 5 | 248 | 0.481 (0.362–0.603) | 60.446 | 1.407 (1.218–1.627) | < 0.001 |
| Particle | 2 | 88 | 0.554 (0.361–0.732) | 0.000 | 1.222 (1.002–1.490) | 0.048 |
| <20 mm |  |  |  |  |  |  |
| RFA | 2 | 99 | 0.851 (0.692–0.936) | 0.000 | 1 | – |
| MWA | 1 | 71 | 0.831 (0.613–0.938) | 0.000 | 1.024 (0.896–1.170) | 0.727 |
| MWA: Microwave ablation; OS: overall survival; RFA: radiofrequency ablation; SABR: stereotactic ablative radiotherapy | | | | | | |
