# Supplementary material for: Comparison of local ablative therapies, including radiofrequency ablation, microwave ablation, stereotactic ablative radiotherapy, and particle radiotherapy, for inoperable hepatocellular carcinoma: a systematic review and meta-analysis
Source: Exp Hematol Oncol. 2023 Apr 12;12:37. doi: 10.1186/s40164-023-00400-7 (PMC10091829; doi:10.1186/s40164-023-00400-7)
Supplement: Supplementary file 10 — Additional file 10: Table S6. Adverse events [file 40164_2023_400_MOESM10_ESM.docx]

| **Additional file 10: Table S6** Adverse events | | | | | | |
| --- | --- | --- | --- | --- | --- | --- |
| Groups | Bleeding | Tumor seeding | Abscess | RILD | dermatitis | hematologic related |
| Local ablative |  |  |  |  |  |  |
| RFA | 0.0% (0/720) | 0.1% (1/720) | 0.0% (0/720) |  |  |  |
| MWA | 0.8% (4/527) | 0.0% (0/527) | 0.2% (1/527) |  |  |  |
| Radiation |  |  |  |  |  |  |
| SABR |  |  |  | 0.7% (3/438) | 0.2% (1/438) | 3.2% (14/438) |
| Particle |  |  |  | 0.0% (0/266) | 0.8% (2/266) | 3.8% (10/266) |
| MWA: Microwave ablation; RFA: radiofrequency ablation; RILD: radiation-induced liver disease; SABR: stereotactic ablative radiotherapy | | | | | | |
