# Supplementary material for: Comparison of local ablative therapies, including radiofrequency ablation, microwave ablation, stereotactic ablative radiotherapy, and particle radiotherapy, for inoperable hepatocellular carcinoma: a systematic review and meta-analysis
Source: Exp Hematol Oncol. 2023 Apr 12;12:37. doi: 10.1186/s40164-023-00400-7 (PMC10091829; doi:10.1186/s40164-023-00400-7)
Supplement: Supplementary file 11 — Additional file 11: Table S7. Average tumor size and percentage of patient with Child-Pugh class B or above in each arm [file 40164_2023_400_MOESM11_ESM.docx]

| **Additional file 11: Table S7** Average tumor size and percentage of patient with Child-Pugh class B or above in each arm | | | | |
| --- | --- | --- | --- | --- |
| Groups | Cohorts (n) | Patients (n) | Average tumor size (mm) | Percentage of patient with Child-Pugh class B or above |
| RFA | 8 | 622 | 35.59 | 0.1141 |
| MWA | 6 | 500 | 27.06 | 0.4640 |
| SABR | 7 | 426 | 41.77 | 0.1373 |
| Particle | 4 | 165 | 50.20 | 0.2222 |
| MWA: Microwave ablation; RFA: radiofrequency ablation; SABR: stereotactic ablative radiotherapy | | | | |
