# Supplementary material for: Comparison of local ablative therapies, including radiofrequency ablation, microwave ablation, stereotactic ablative radiotherapy, and particle radiotherapy, for inoperable hepatocellular carcinoma: a systematic review and meta-analysis
Source: Exp Hematol Oncol. 2023 Apr 12;12:37. doi: 10.1186/s40164-023-00400-7 (PMC10091829; doi:10.1186/s40164-023-00400-7)
Supplement: Supplementary file 12 — Additional file 12: Table S8. Methodological quality assessment of randomized controlled trials [file 40164_2023_400_MOESM12_ESM.doc]

| **Additional file 12: Table S8** Methodological quality assessment of randomized controlled trials | | | | | | |
| --- | --- | --- | --- | --- | --- | --- |
| Study  [year] | Bias arising from the randomisation process | Bias due to deviations from intended interventions | Bias due to missing outcome data | Bias in outcome measurement | Bias in the selection of the reported result | Overall risk of bias |
| Abdelaziz et al. [2014] | Some concerns^a^ | Some concerns^b^ | Low risk | High risk^c^ | Low risk | High risk |
| Chong et al. [2020] | Low risk | Some concerns^b^ | Low risk | Low risk | Low risk | Some concerns |
| Kan et al. [2015] | Some concerns^a^ | Low risk | Low risk | Low risk | Low risk | Some concerns |
| Nojiri et al. [2017] | Low risk | Low risk | Low risk | Low risk | Low risk | Low risk |
| Tak et al. [2018] | Low risk | Low risk | Low risk | Low risk | Low risk | Low risk |
| Violi et al. [2018] | Low risk | Some concerns^b^ | Low risk | Low risk | Low risk | Some concerns |

Methodological quality assessment was based on the Cochrane risk of bias tool (RoB 2.0).

^a^Lack of information on the concealment of the allocation sequence.

^b^Lack of information on blinding

^c^Lack of information on outcome assessor blinding
