# Supplementary material for: Comparison of local ablative therapies, including radiofrequency ablation, microwave ablation, stereotactic ablative radiotherapy, and particle radiotherapy, for inoperable hepatocellular carcinoma: a systematic review and meta-analysis
Source: Exp Hematol Oncol. 2023 Apr 12;12:37. doi: 10.1186/s40164-023-00400-7 (PMC10091829; doi:10.1186/s40164-023-00400-7)
Supplement: Supplementary file 13 — Additional file 13: Table S9. Methodological quality assessment of nonrandomized studies [file 40164_2023_400_MOESM13_ESM.doc]

| **Additional file 13: Table S9** Methodological quality assessment of nonrandomized studies | | | | | | | | |
| --- | --- | --- | --- | --- | --- | --- | --- | --- |
| Study  [year] | Pre-intervention | | Intervention | Post-intervention | | | | Overall |
|  | Bias due to confounding | Bias in selection | Bias in classification of interventions | Bias from intended interventions | Bias due to missing data | Bias in measurement of outcomes | Bias in selection of the reported result | Overall bias |
| Bujold et al.  [2013] | Low | Low | Low | Moderate | Low | Low | Low | Moderate |
| Choi et al.  [2016] | Moderate | Low | Low | Low | Low | Low | Low | Moderate |
| Cillo et al.  [2014] | Low | Low | Low | Low | Low | Low | Low | Low |
| Darweesh et al.  [2019] | Moderate | Low | Low | Low | Low | Low | Low | Moderate |
| Durand-Labrunie et al. [2020] | Low | Low | Low | Low | Low | Low | Low | Low |
| Feng et al.  [2018] | Low | Low | Low | Low | Low | Low | Low | Low |
| Francica et al.  [2019] | Moderate | Low | Low | Moderate | Low | Low | Low | Moderate |
| Imada et al.  [2010] | Moderate | Low | Low | Low | Low | Low | Low | Moderate |
| Kimura et al.  [2017] | Moderate | Low | Low | Low | Low | Low | Low | Moderate |
| Kimura et al.  [2021] | Moderate | Low | Low | Low | Low | Low | Low | Moderate |
| Lasley et al.  [2015] | Low | Low | Moderate | Moderate | Low | Low | Low | Moderate |
| Liu et al.  [2017] | Moderate | Low | Low | Low | Low | Low | Low | Moderate |
| Nakayama et al.  [2011] | Moderate | Moderate | Low | Low | Low | Low | Low | Moderate |
| Parzen et al.  [2020] | Moderate | Moderate | Low | Low | Low | Low | Low | Moderate |
| Scorsetti et al.  [2015] | Moderate | Low | Low | Low | Low | Low | Low | Moderate |
| Wang et al.  [2010] | Low | Low | Low | Low | Low | Low | Low | Low |
| Weiner et al.  [2016] | Moderate | Low | Low | Low | Low | Low | Low | Moderate |
| Yao et al.  [2021] | Low | Low | Low | Low | Low | Low | Low | Low |
| Yu et al.  [2018] | Moderate | Moderate | Low | Low | Low | Low | Low | Moderate |
| Zhou et al.  [2011] | Moderate | Low | Low | Moderate | Low | Low | Low | Moderate |
