# Supplementary material for: Comparison of local ablative therapies, including radiofrequency ablation, microwave ablation, stereotactic ablative radiotherapy, and particle radiotherapy, for inoperable hepatocellular carcinoma: a systematic review and meta-analysis
Source: Exp Hematol Oncol. 2023 Apr 12;12:37. doi: 10.1186/s40164-023-00400-7 (PMC10091829; doi:10.1186/s40164-023-00400-7)
Supplement: Supplementary file 14 — Additional file 14: Heterogeneity and Limitation [file 40164_2023_400_MOESM14_ESM.docx]

**Additional file 14:** Heterogeneity and Limitation

**Heterogeneity**

The heterogeneity varied in our study, with most of the comparisons being at low to moderate levels while the 4-year overall survival rate in stereotactic ablative radiotherapy (SABR) was at an extremely high level. Various factors may cause the heterogeneity. First, the time of ablation in studies was different. Second, the SABR and particle radiotherapy may differ due to the manual manipulation of the instrument, thereby impacting the radiation zone and the treatment effect. Third, we evaluated inoperable patients with hepatocellular (HCC). The reason for their inoperability varied and might impact heterogeneity. Fourth, the tumor size varied among studies, and its impact on the HCC staging and the progression and overall survival rate might be different. The operator’s skills also caused heterogeneity, especially in radiofrequency ablation therapy (RFA) and microwave ablation therapy (MWA).

**Limitation**

Our study had some limitations. First, the studies that focused on particle therapy were relatively less than RFA and MWA. Second, particle therapy includes proton and carbon ion therapy which are different in biological effect, but it is difficult to separate the results between proton and carbon ion therapy due to the small amount and small sample size of prospective studies in these therapies. Third, the included studies were mainly Child-Pugh class A and single nodule. Fourth, although we determine to perform assessment of publication bias in the method section, we did not perform the assessment of publication bias because data would be unreliable in view of the few studies (less than 10) included for each treatment group (1). Last, although our study confirmed MWA, SABR and particle radiotherapy were effective alternatives to RFA for inoperable HCC, we thought a more comprehensive clinical trial to compare the above treatment strategies was needed. Considering the difficulty of conducting these comparisons in real world, carrying out cell experiment to verify the patient’s outcome were further explored (2, 3). Besides, after obtaining more real world data, building a prognostic model may assist to assist physicians develop a personalized treatment, promoting patient’s life quality and lowering the potential harmful effect (4).

Reference

1. Ren X, Zhang T, Chen X, Wei X, Tian Y, Li G, et al. Early-life exposure to bisphenol A and reproductive-related outcomes in rodent models: a systematic review and meta-analysis. Aging (Albany NY). 2020; 12(18): 18099.
2. Ye S, Liu Q, Huang K, Jiang X, Zhang X. The comprehensive analysis based study of perfluorinated compounds—Environmental explanation of bladder cancer progression. Ecotoxicology and Environmental Safety. 2022; 229: 113059.
3. Zhang T, Wu J, Zhang X, Zhou X, Wang S, Wang Z. Pharmacophore based in silico study with laboratory verification—environmental explanation of prostate cancer recurrence. Environmental Science and Pollution Research. 2021; 28: 61581-61591.
4. Liu Y, Wang J, Li L, Qin H, Wei Y, Zhang X, et al. AC010973. 2 promotes cell proliferation and is one of six stemness-related genes that predict overall survival of renal clear cell carcinoma. Scientific Reports. 2022; 12(1): 4272.
